# Enhanced Hydrogen Evolution Reaction Using Biomass-Activated Carbon Nanosheets Derived from Eucalyptus Leaves

**DOI:** 10.3390/ma18030670

**Published:** 2025-02-03

**Authors:** Sankar Sekar, Atsaya Shanmugam, Gokilapriya Senthilkumar, Kiruthiga Thangasami, Hyun Jung, Youngmin Lee, Sejoon Lee

**Affiliations:** 1 Division of System Semiconductor, Dongguk University-Seoul, Seoul 04620, Republic of Korea; sanssekar@dongguk.edu (S.S.); atsyshanmu@dgu.ac.kr (A.S.); priya@dgu.ac.kr (G.S.); 2 Quantum-Functional Semiconductor Research Center, Dongguk University-Seoul, Seoul 04620, Republic of Korea; 3 Advanced Functional Nanohybrid Material Laboratory, Department of Chemistry, Dongguk University Seoul, Seoul 04620, Republic of Korea; kiruthigat22@dgu.ac.kr (K.T.); chemphile@dongguk.edu (H.J.); 4 Department of Advanced Battery Convergence Engineering, Dongguk University Seoul, Seoul 04620, Republic of Korea

**Keywords:** eucalyptus leaves, activated carbon, biomass, electrocatalysts, hydrogen evaluation reaction

## Abstract

Carbonaceous-based metal-free catalysts are promising aspirants for effective electrocatalytic hydrogen generation. Herein, we synthesized mesoporous-activated carbon nanosheets (ELC) from biomass *eucalyptus* leaves through KOH activation. The microstructure, structural, and textural characteristics of the prepared materials were characterized by FE-SEM, Raman, XRD, and BET measurements. The high temperature (700 °C) KOH-activated ELC nanosheets exhibited an interconnected nanosheet morphology with a large specific surface area (1436 m^2^/g) and high mesoporosity. The ELC-700 catalyst exhibited an excellent electrocatalytic HER performance with a low overpotential (39 mV at 10 mA/cm^2^), excellent durability, and a Trivial Tafel slope (36 mV/dec) in 0.5 M H_2_SO_4_ electrolyte. These findings indicate a new approach for developing excellent biomass-derived electrocatalysts for substantially efficient green hydrogen production.

## 1. Introduction

Environmental changes and global warming driven by the utilization of fossil fuels have intensified substantial interest in pursuing sustainable, clean, and green renewable energy sources [1,2,3,4]. Green hydrogen is considered one of the most versatile alternative energy sources due to its zero-carbon emission, sustainability, eco-friendliness, and excellent energy density [5,6,7,8]. Among the various production techniques, electrocatalytic water splitting is the most efficient, clean, and scalable approach for producing green hydrogen via hydrogen evolution reaction (HER) [9,10,11,12]. However, the insignificant reaction kinetics and the high overpotential of HER restrict the catalytic efficiency of water splitting [13,14]. Consequently, developing highly efficient electrocatalysts is essential to lower the energy barrier and accelerate hydrogen production. Currently, platinum (Pt)-based derivates are used as efficient catalysts for HER, but their shortage and expensiveness impede their large-scale industrial application [15,16,17]. Recently, various alternatives have been classified as promising high-performance HER electrocatalysts to replace Pt-based catalysts. For instance, the transition of metal dichalcogenides [18,19,20], noble metal alloys [21,22], carbides [23,24], phosphides [25,26], oxides [27,28,29], nitrides [30], metal–organic frameworks [31,32,33], single-atom catalysts [34], layered double hydroxides [35,36], and perovskite- [37] and spinel-structured materials [8,38] are distinctive catalysts that exhibit excellent electrocatalytic HER performances. However, due to complex and expensive synthesis methods, insignificant electrical conductivity, and poor durability, efforts have been made to avoid using metal-based electrocatalysts [39]. Consequently, developing inexpensive, highly effective, metal-free, and acid-stable HER catalysts from cost-effective natural resources is vital.

Currently, different kinds of carbonaceous materials (e.g., porous carbon [40,41,42], graphitic carbon [11,43], carbon fiber [44], activated carbon (AC) [45,46,47], carbon nanotube [48], graphene oxide [49], and graphene [15,50,51]) have been investigated as HER electrocatalysts. Owing to the large textural area, natural abundance, high conductivity, low cost, tunable porous structure, and robust stability, biomass-derived AC has attracted significant attention as HER electrocatalysts [40,41,42,52]. For example, Liu et al. [40] synthesized N,S-doped porous carbon nanosheets from human hair using ZnCl_2_ activation under a nitrogen atmosphere. These nanosheets exhibited excellent HER performance, achieving a small overpotential of 12 mV with remarkable durability. Cao et al. [41] fabricated N-porous carbon from bean sprouts using a SiO_2_ template method and demonstrated the overpotential of 413 mV with a Tafel value of 98 mV/dec. Sun et al. [45] used waste paper for preparing Co and N co-doped carbon through hydrothermal and pyrolysis methods, retaining a low overpotential of 223 mV at 10 mA/cm^2^. Prabu et al. [42] prepared hierarchical porous carbon nanosheets from palm plants and showed an overpotential of 330 mV with a Tafel value of 63 mV/dec. Among various biomass resources, *eucalyptus* leaves have attracted attention as a sustainable biomass precursor for the fabrication of AC nanostructures due to their cheap, natural abundance, environmental friendliness, and high carbon content (~75%) [53,54,55,56]. Therefore, salvaging abundant biomass *eucalyptus* leaves can be a favorable material for preparing high-performance AC nanostructures. Despite such benefits, no studies on biomass *eucalyptus* leaves-derived mesoporous AC (ELC) nanostructures for HER electrocatalysts have been reported so far.

Despite all the above, herein, we fabricated mesoporous AC nanosheets from biomass *eucalyptus* leaves by using the KOH activation process at 600–700 °C. The ELC-700 catalyst exhibited a small overpotential of 39 mV with a low Tafel value of 36 mV/dec at 10 mA/cm^2^ in 0.5 M H_2_SO_4_. The material preparation, material characterization, textural characteristics, and the electrocatalytic HER activities of the prepared catalysts were systematically evaluated and deliberated in detail.

## 2. Experimental Section

### 2.1. Preparation of ELC Nanosheets

**Figure 1:**
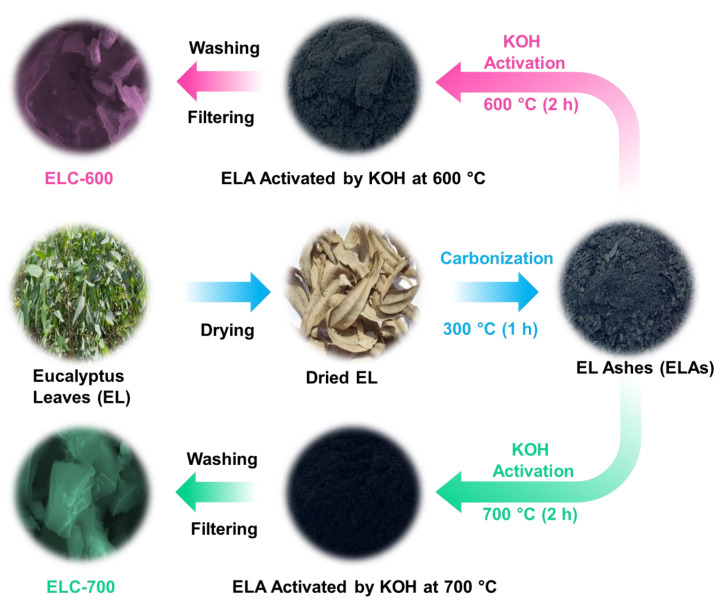
Schematic description of the experimental procedure for ELC-600 and ELC-700 nanosheets from eucalyptus leaves through the KOH activation.

Figure 1 illustrates the schematic process for synthesizing ELC nanosheets. Biomass *eucalyptus* leaves (EL) were collected from Tholudur, Tamil Nadu, India, and served as the carbonaceous precursor. Initially, the EL was washed with deionized (DI) water and air-dried for five days. Then, the parched ELs were carbonized at 300 °C for 60 min in a muffle furnace to produce carbonized ashes (ELAs). After carbonization, the ELA-KOH mixture was prepared using ELA (4 g) and KOH (16 g) in a mortar. Next, the prepared mixture was stimulated at two various temperatures (600 and 700 °C) for 120 min in an air atmosphere. During the KOH activation, oxygen- and carbon-containing groups react with KOH; then, carbonaceous chemicals (e.g., carbon monoxide (CO), potassium carbonate (K_2_CO_3_), etc.) are formed. The activation process was carried out at high temperatures and is illustrated by the following chemical reactions [57,58]:

(1) $$6KOH+2C\left(ELAs\right)\to 2K+2K_{2}CO_{3}+3H_{2}$$

(2) $$K_{2}CO_{3}\to K_{2}O+CO_{2}$$

(3) $$C+CO_{2}\to 2CO$$

(4) $$K_{2}CO_{3}+2C\to 2K+3CO$$

(5) $$C+K_{2}O\to 2K+CO$$

Next, the KOH-treated ELA was blended in DI water (100 mL) and stirred for 720 min to eliminate unreacted potassium components. After that, the soaked colloidal mixture was filtered, rinsed, and dried at 150 °C for 720 min. Finally, the powder form of ELC nanosheets was observed and labeled as ‘ELC-600’ and ‘ELC-700’ for KOH-activated at 600 and 700 °C, respectively.

### 2.2. Physicochemical Characterizations

The surface morphology of the prepared ELC-600 and ELC-700 catalysts was inspected by using scanning electron microscopy (FE-SEM, Clara LMH, Tescan Brno, Brno-Kohoutovice, Czech Republic). The vibrational and structural characteristics of the fabricated catalysts were assessed using Raman scattering spectroscopy (LabRAM HR800, Jobin Yvon, Longjumeau, France) and X-ray diffraction (XRD, D8-Advance, Bruker, Madison, WI, USA), respectively. The porosity and textural characteristics were investigated using Barrett–Joyner–Halenda (BJH) and Brunauer–Emmett–Teller (BET) measurements (BELSORP-mini II system, MicrotracBEL, Osaka, Japan).

### 2.3. Electrochemical HER Measurements

The electrocatalytic HER performances of the ELC-600 and ELC-700 catalysts were assessed using a VersaSTAT3 workstation (Ametek Scientific Company, Berwyn, PA, United States of America) with a typical three-electrode system. Initially, we assembled the two working electrodes using activated carbon (ELC-600 or ELC-700) mixed with an N-methyl-2-pyrrolidinone solvent. After that, the mixed slurries were smeared on the stainless-steel substrates (1 cm × 1 cm) and desiccated at 160 °C for 720 min. Additionally, the saturated calomel electrode (SCE) and the coiled Pt wire functioned as the reference and counter electrodes, respectively. All the HER measurements (i.e., linear sweep voltammetry (LSV), chronopotentiometry (CP), cyclic voltammetry (CV), and electrochemical impedance spectroscopy (EIS)) were conducted in 0.5 M H_2_SO_4_. CV characteristics were obtained at various current densities (10–100 mV/s) with a window range of 0–1.0 V. LSV characteristics of the ELC-600 and ELC-700 catalysts were examined in the window range of 0.1 to 1.2 V at 5 mV/s. The HER rate performances were evaluated via CP measurements at several current densities of -10 to 100 mA/cm^2^ for 10 min. EIS characteristics were assessed at a frequency range of 1 Hz to 10 kHz. Electrochemical double-layer capacitances (*C_dl_*) of the fabricated catalysts were calculated from the non-Faradic CV region to assess the electrochemically active surface area (*ECSA*) using the following equations [8,59,60,61]:

(6) $$J_{DL}=C_{dl}\times v/A$$

(7) $$ECSA=C_{dl}/C_{e}$$

where A is the fabricated electrode area, J_DL_ is the non-Faradaic current density, C_e_ is the capacitance of the electrolyte (1 M H_2_SO_4_ = 0.035 mF/cm^2^), *v* is the scan rate, and C_dl_ is the non-Faradaic capacitance. The overpotential (*η*) and the Tafel slope (*S*_T_) values of the fabricated catalysts were derived using the following equations [11,15,46]:

(8) $$E_{RHE}=E_{SCE}+0.059\cdot pH+E_{SCE}^{0}$$

(9) $$E_{RHE}=\eta$$

(10) $$\eta =S_{T}\log\left(J\right)+c$$

where *c*, *E^0^_SCE_*, *E_RHE_*, and *J* are the fitting parameters, SCE’s standard potential, RHE’s standard potential, and the current density, respectively.

## 3. Results and Discussion

**Figure 2:**
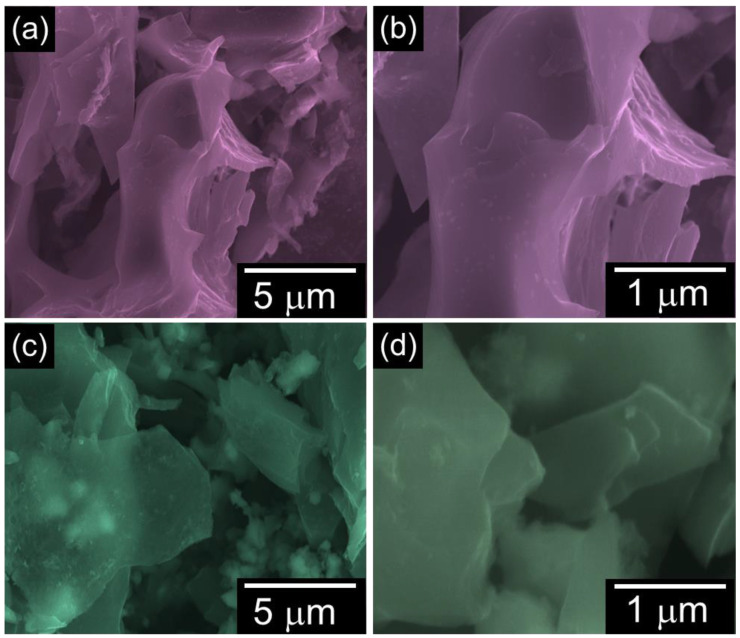
Low- and high-magnification FE-SEM images of (**a**,**b**) ELC-600 and (**c**,**d**) ELC-700 nanosheets.

The topography insights of the biomass EL-derived ELC-600 and ELC-700 were inspected via FE-SEM measurement. Figure 2 displays the FE-SEM images of the prepared catalysts. The ELC-600 sample exhibits irregular and aggregated stacked sheets-like morphology (Figure 2a,b). Conversely, the ELC-700 clearly shows a structure of agglomerated and interconnected nanosheets (Figure 2c,d). However, the interconnected nanosheet textures are clearly more visible in ELC-700 than in ELC-600. This structural enhancement in ELC-700 could potentially improve the catalytic active sites and porosity, which is beneficial for enhancing the HER activities. The EDX analysis (see Figure S1) revealed that both samples predominantly consisted of carbon, confirming the high purity of the materials.

**Figure 3:**
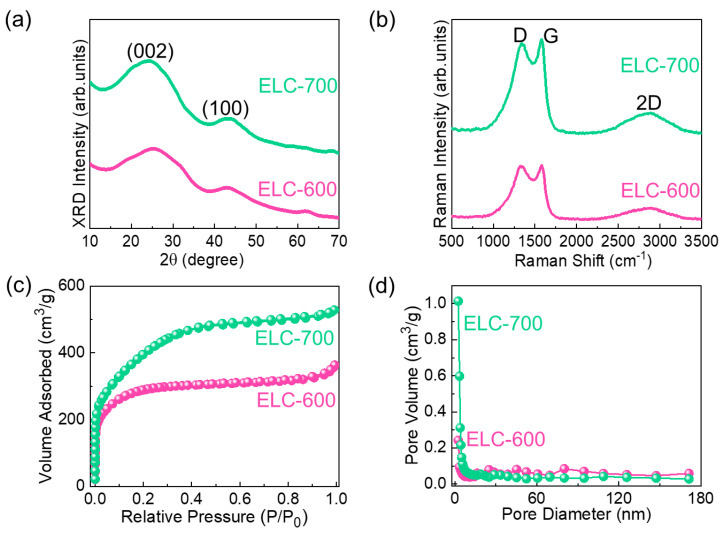
(**a**) XRD patterns, (**b**) Raman spectra, (**c**) Nitrogen absorption–desorption isotherms, and (**d**) Pore size distributions of ELC-600 and ELC-700 nanosheets.

The structural characteristics of the ELC-600 and ELC-700 samples were investigated by XRD measurements. Figure 3a demonstrates the XRD pattern of the ELC-600 and ELC-700. Both materials exhibited the two diffraction peaks at 24.2° and 43.5° are associated with the (002) and (100) crystalline planes of the disordered carbon structure with the amorphous nature of the materials [57,62,63,64], respectively. Compared to ELC-700, the ELC-600 sample exhibited broader (002) diffraction peaks, demonstrating a high degree of disordered structure. This result indicates that as the KOH activation temperature increases, the carbon materials become more graphitic, which enhances the electrical conductivity of the materials [65]. No other impurities were observed, suggesting the high purity of the samples. Furthermore, the disordered structures of the carbonaceous materials were investigated by Raman measurements. Figure 3b illustrates the Raman spectra of the ELC-600 and ELC-700 samples. Both samples indicate three characteristic peaks at 1346 cm^−1^ (D), 1579 cm^−1^ (G), and 2889 cm^−1^ (2D), demonstrating the graphitic carbon nature of the prepared materials [11,53,66]. The D band is accompanied by the disorder vibration with dangling bonds in terminal planes (A1g symmetry) of graphitic carbon [67,68]. G band is ascribed to the E_2g_ vibration mode of the sp^2^ hybridized carbon electronic configuration [40]. The 2D band acts as a typical signature of activated carbon [57,69]. The band intensity ratio (I_D_/I_G_) of the ELC-600 and ELC-700 samples were determined to be 0.99 and 0.97, respectively. All of the above indicates that, compared to ELC-600, the ELC-700 sample exhibits a trivial disordered lattice structure and a high level of graphitization. Furthermore, the surface functional characteristics of the ELC-600 and ELC-700 samples were investigated by FTIR instruments (Figure S2). The wider peak in the region of 3010–3647 cm^−1^ is attributed to the O-H stretching vibrations because of the vibrations of the water molecules or surface-adsorbed moisture [47]. The peak at 2916 cm^−1^ is correlated with the C-H stretching vibrations (i.e., CH_3_, CH_2_, and CH groups) [70]. The peaks at 2356, 1553, and 986 cm^−1^ are correlated to the C = O, C = C, and C-C stretching vibration of the activated carbon, respectively [46,71,72]. While increasing the activation temperature, the water molecules or surface-adsorbed moisture are reduced due to modified hydrogen bonding networks, and the samples showed a slight intensity and peak shift in the FTIR spectra.

The textural characteristics and the porosity nature of the ELC-600 and ELC-700 samples were investigated by BET and BJH measurements. As displayed in Figure 3c, both materials distinctly display a Type-IV physisorption isotherm (verified by IUPAC), demonstrating the mesoporous characteristics of the AC nanosheets [15,57,64,66,67]. However, the coinciding adsorption and desorption branches accentuate the absence of a hysteresis loop, suggesting a uniform and adequate pore structure without significant capillary condensation effects. From the BET measurements, the surface area of the ELC-600 and ELC-700 were estimated to be 1022 and 1436 m^2^/g, respectively. Figure 3d depicts the pore characteristics of the ELC-600 and ELC-700. The pore surface area of ELC-600 and ELC-700 are 132 and 507 m^2^/g, respectively. In addition, the total pore volume of the ELC-700 (0.5122 cm^3^/g) sample is higher than that of the ELC-600 (0.3358 cm^3^/g) sample. In addition, the average pore size of the ELC-600 and ELC-700 samples was estimated to be 2.27 and 2.18 nm, respectively. From the t-plot (Figure S3), we calculated the mesoporous surface area of the ELC-600 and ELC-700 are 217 and 433 m^2^/g, respectively. The micropore volumes of the ELC-600 and ELC-700 were determined to be 0.028 and 0.036 cm^3^/g, respectively. Compared to ELC-600 (0.307 cm^3^/g), the ELC-700 sample (0.476 cm^3^/g) exhibited a high mesopore volume, indicating the high mesoporosity of the ELC-700 material. This result clearly indicates that the ELC-700 sample has a high mesoporosity nature with significant micropores compared to ELC-600, which could be favorable for high electrocatalytic HER performance.

**Figure 4:**
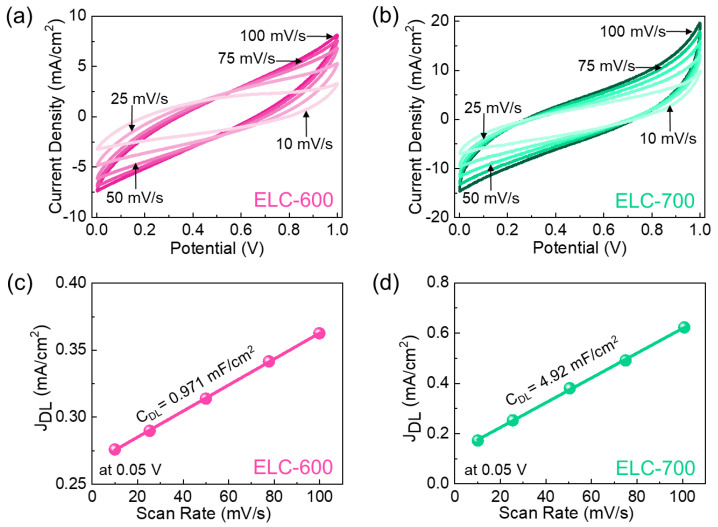
CV curves of the (**a**) ELC-600 and (**b**) ELC-700 catalysts. Non-Faradaic *J*_DL_ at 0.05 V as a function of the scan rate of the (**c**) ELC-600 and the (**d**) ELC-700 catalysts.

After confirming the material characteristics of the prepared catalysts, we evaluated the electrocatalytic HER performances of ELC-600 and ELC-700 catalysts. Figure 4a,b show the CV characteristics of the ELC-600 and ELC-700 catalysts at different scan rates of 10–100 mV/s. Both catalysts showed distinctive rectangular-shaped CV curves, indicating the effective electrical double-layer capacitive behavior of the materials [11,53,56]. When the scan rate rose, the current density also improved owing to the small diffusion resistance of the active catalysts. Compared to the ELC-600, the ELC-700 catalyst exhibited a substantial CV loop area and a higher current response that demonstrated the ELC-700 has more active sites than the ELC-600 catalyst. The electrocatalytic HER activities are correlated with the electrochemical double-layer capacitance (*C_dl_*) and electrochemically active surface area (*ECSA*) of the catalyst material. The *C_dl_* values of the ELC-600 and ELC-700 catalysts were calculated to be 0.971 and 4.92 mF/cm^2^ from the non-Faradic CV region (0.05V, see Figure 4c,d, and Figure S4), respectively. The ECSA values are 33 cm^2^ (ELC-600) and 158 cm^2^ (ELC-700) using Equations (6) and (7). Compared to ELC-600, the ELC-700 catalyst has higher *C_dl_* and ECSA values, demonstrating the ELC-700 revealed larger catalytic active sites and higher electrical conductivity.

**Figure 5:**
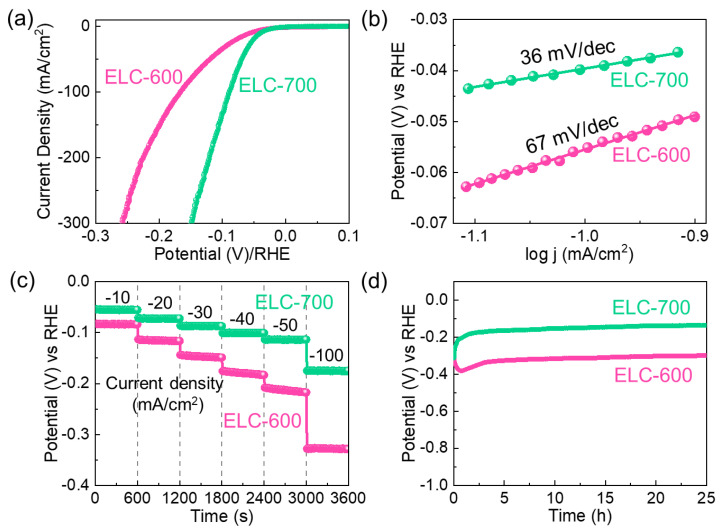
HER activities of the ELC-600 and ELC-700 catalysts. (**a**) *i_R_*-corrected LSV curves, (**b**) Tafel plots, (**c**) chronopotentiometric profiles at different current densities (−10 to −100 mA/cm^2^), and (**d**) long-term stability characteristics.

The ELC-600 and ELC-700 catalysts’ HER performances were assessed using an LSV measurement conducted at 5 mV/s in a 0.5 M H_2_SO_4_ acidic solution. Figure 5a depicts the *i_R_*-corrected LSV slopes of the ELC-600 and ELC-700 catalysts. The *η* values of the ELC-600 and ELC-700 catalysts were measured to be 55 mV and 39 mV at 10 mA/cm^2^ (from Equations (8) and (9)), respectively. The ELC-700 catalyst exhibited lower *η* values due to its larger catalytic-active sites (i.e., higher ECSA) and higher conductivity than the ELC-600 catalyst. As shown in Figure 5b, the S_T_ value of the ELC-600 and ELC-700 catalysts were measured to be 67 and 36 mV/dec, respectively, by utilizing Equation (10). The lower S_T_ value of the ELC-700 catalyst exhibited the more efficient HER kinetics ensuing the Volmer–Heyrovsky mechanism. Therefore, it is widely recognized that the HER mechanism unfolds on the cathode surface through a multistep electrochemical reaction process. Specifically, in an acidic electrolyte medium, the multistep HER process proceeds via the following reaction steps [73,74].

(11) $$H_{3}O^{+}+e^{-}+M\to M-H+H_{2}O\;(Volumer)$$

(12) $$H_{3}O^{+}+M-H+e^{-}\to H_{2}O+H_{2}+M(Heyrovsky)$$

(13) $$2M-H\to 2M+H_{2}(Tafel)$$

where the M-H and M are related to the absorbed hydrogen atoms and vacant surface site of the catalysts. The strength of the M-H bond is unanimously critical for determining the HER kinetics of catalyst materials [75]. Compared to the other electrocatalysts, the ELC-700 catalyst exhibited excellent HER activities (i.e., lower *η* and S_T_ values) because of the higher ECSA and high conductivity (Table S1).

The superb HER performances of the ELC-600 and ELC-700 catalysts were further clarified by using CP measurements. Figure 5c shows the CP characteristics of the ELC-600 and ELC-700 catalysts. The CP measurements clearly show that the ELC-700 catalyst has a lower overpotential at each current density (i.e., −10, −20, −30, −40, −50, and −100 mA/cm^2^) than that of the ELC-600 catalyst. Figure 5d indicates the long-term HER stability measurements of the catalysts at −10 mA/cm^2^ for 25 h. Compared to the ELC-600 catalyst, the ELC-700 catalyst demonstrated stable and long-term HER stability because of the high ECSA, large porosity, and low resistance of the material. Moreover, the LSV slopes of the catalysts are nearly the same before and after the long-term stability test (Figure S5). These indicate that the ELC-700 catalyst can serve as a stable and excellent electrocatalyst for HER. After the HER stability, we carried out the FE-SEM, Raman, and FTIR measurements to examine the changes in both the microstructural and vibrational characteristics of the fabricated catalysts. From FE-SEM measurements, the ELC-600 catalyst exhibited the aggregated sheet structure (see Figure S6a). However, the ELC-700 catalyst still maintained its original agglomerate nanosheet structure (see Figure S6b). From Raman measurements (see Figure S7), both catalysts still maintain their original nature, indicating the highly stable nature of the materials. Furthermore, the FTIR spectra (see Figure S8) of both catalysts clearly exhibited the original structural vibration of the material. Compared to the ELC-700 catalyst, the FTIR spectra of the ELC-600 catalyst are slightly intensity and peak shift demonstrating the ELC-700 catalyst has a high stable nature of the materials.

**Figure 6:**
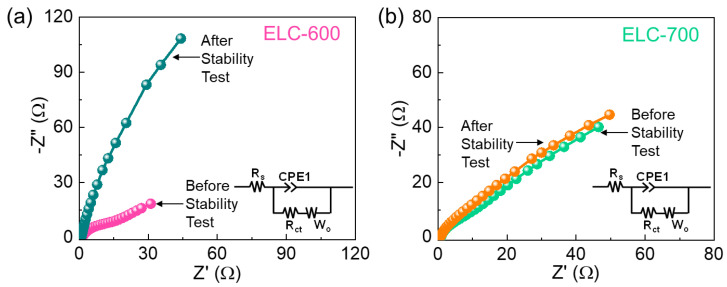
Nyquist plots of (**a**) ELC-600 and (**b**) ELC-700 catalysts before and after the stability test (Inset in the equivalent circuit of the active catalysts).

To further clarify the enhanced HER kinetics of the ELC-700 catalyst, EIS measurements were conducted. Figure 6 displays the Nyquist plot of the ELC-600 and ELC-700 catalysts with their subsequent equivalent circuit (Figure 6, inset). Both catalysts displayed a linear line at lower frequencies due to the distribution of the ionic solution across the catalyst surface [76,77]. However, the absence of a semicircle in both catalysts indicates the high ionic diffusivity and extraordinary electronic conductivity [78,79]. The serial resistance (R_s_) values of the ELC-600 and ELC-700 catalysts are 1.27 Ω and 0.85 Ω, respectively, by using an equivalent circuit model. Compared to the ELC-600 catalyst, the ELC-700 catalyst showed a low R_s_ value and steeper slope because of the high active sites, large porosity, enhanced ionic diffusion, and high conductivity. From all the above results, the biomass-derived ELC-700 nanosheets are a more sustainable catalyst material for excellent HER electrocatalyst for future green hydrogen production.

## 4. Conclusions

Mesoporous EL-AC nanosheets were effectively derived from biomass *eucalyptus* leaves via KOH activation. The ELC-700 nanosheets exhibited mesoporous agglomerated and interconnected nanosheet morphology with a high specific textural area. Owing to the mesoporous nature and the superior graphitization of ELC-700, the catalyst revealed excellent HER performances with a significantly smaller *η* (i.e., 39 mV at 10 mA/cm^2^) and a lower S_T_ (i.e., 36 mV/dec) in a 0.5 M H_2_SO_4_ solution, compared to those of ELC-700 (i.e., η: 55 mV and S_T_: 67 mV/dec). These outcomes indicate that mesoporous EL-AC nanosheets hold promise as a tremendous HER electrocatalyst for future green hydrogen production.

## Data Availability

No data were used for the research described in the article.

## Supplementary Materials

The following supporting information can be downloaded at https://www.mdpi.com/article/10.3390/ma18030670/s1. Figure S1: EDX spectra of the (a) ELC-600 and (b) ELCC-700 samples; Figure S2: FTIR spectra of the ELC-600 and ELC-700 samples; Figure S3: t-plot of the ELC-600 and ELC-700 samples; Figure S4: Non-Faradaic CV curves at 0.05 V of the (a) ELC-600 and (b) ELC-700 catalysts; Figure S5: LSV curves of the (a) ELC-600 and (b) ELC-700 catalysts before and after the HER stability test; Figure S6: FE-SEM images of the (a) ELC-600 and the (b) ELC-700 catalysts after the stability test; Figure S7: Raman spectra of the (a) ELC-600 and the (b) ELC-700 catalysts after the stability test; Figure S8: FTIR spectra of the (a) ELC-600 and the (b) ELC-700 catalysts after the stability test; Table S1: Comparison of the HER performances between biomass ELC nanosheets and other carbonaceous electrocatalysts reported in previous works.
