# Supplementary material for: Enhanced Hydrogen Evolution Reaction Using Biomass-Activated Carbon Nanosheets Derived from Eucalyptus Leaves
Source: Materials (Basel). 2025 Feb 3;18(3):670. doi: 10.3390/ma18030670 (PMC11820768; doi:10.3390/ma18030670)
Supplement: Supplementary file 1 [file materials-18-00670-s001.zip › materials-3345180-supplementary.pdf]

Supplementary Materials

Enhanced Hydrogen Evolution Reaction Using Biomass-Activated Carbon Nanosheets Derived from Eucalyptus Leaves

Sankar Sekar [1,2], Atsaya Shanmugam [1,2], Gokilapriya Senthilkumar [1,2], Kiruthiga Thangasami [3,4], Hyun Jung [3,4], Youngmin Lee [1,2,]* and Sejoon Lee [1,2,]*

1 Division of System Semiconductor, Dongguk University-Seoul, Seoul 04620, Republic of Korea;
sanssekar@dongguk.edu (S.S.); atsyshanmu@dgu.ac.kr (A.S.); priya@dgu.ac.kr (G.S.)
2 Quantum-Functional Semiconductor Research Center, Dongguk University-Seoul, Seoul 04620,
Republic of Korea
3 Advanced Functional Nanohybrid Material Laboratory, Department of Chemistry, Dongguk University Seoul, Seoul 04620, Republic of Korea; kiruthigat22@dgu.ac.kr (K.T.); chemphile@dongguk.edu (H.J.)
4 Department of Advanced Battery Convergence Engineering, Dongguk University Seoul, Seoul 04620,
Republic of Korea
* Correspondence: ymlee@dongguk.edu (Y.L.); sejoon@dongguk.edu (S.L.)

■ Elemental Characteristics of ELC-600 and ELC-700

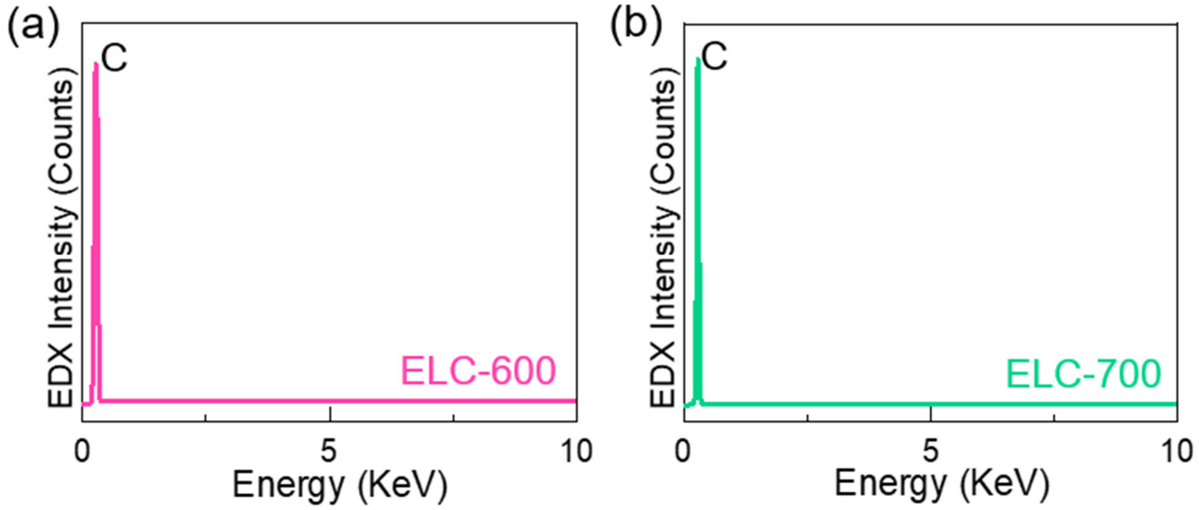

Figure S1: EDX spectra of the (a) ELC-600 and (b) ELCC-700 samples.

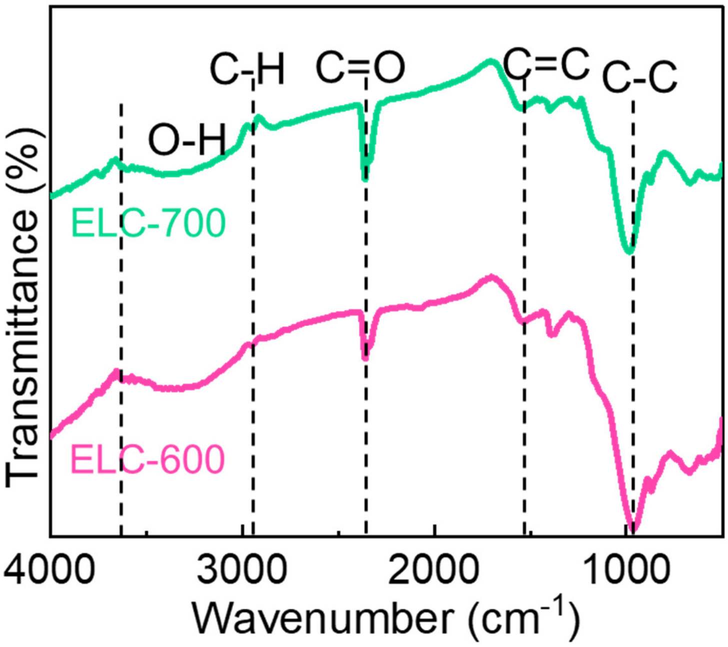

Figure S2: FTIR spectra of the ELC-600 and ELC-700 samples.

■ Textural Properties of ELC-600 and ELC-700

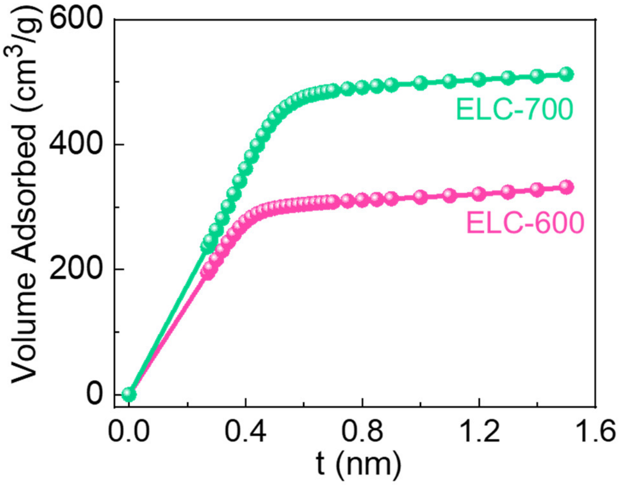

Figure S3: t-plot of the ELC-600 and ELC-700 samples.

■ Electrochemical Properties of ELC-600 and ELC-700

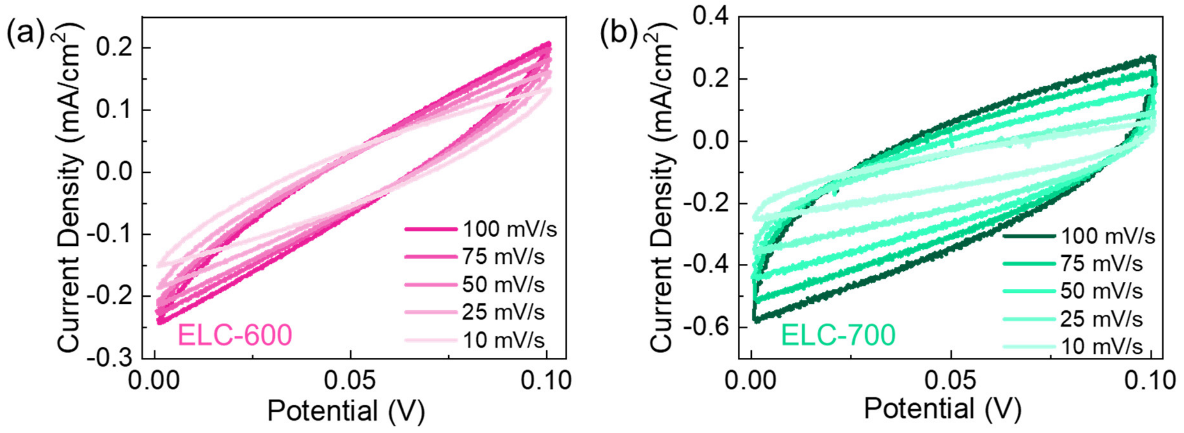

Figure S4: Non-Faradaic CV curves at 0.05 V of the (a) ELC-600 and (b) ELC-700 catalysts.

■ Electrocatalytic Properties of ELC-600 and ELC-700 before and after Stability Test

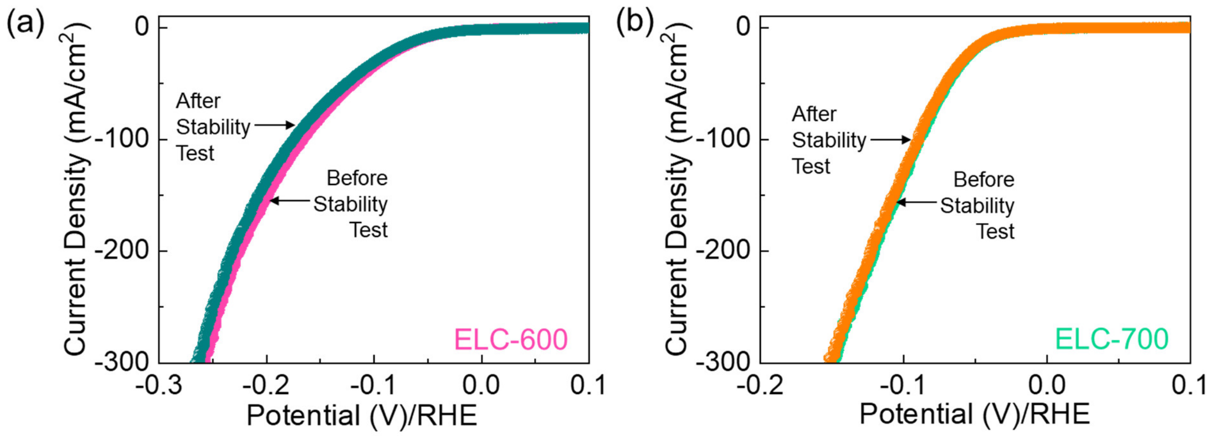

Figure S5: LSV curves of the (a) ELC-600 and (b) ELC-700 catalysts before and after the HER stability test.

■ **Morphological Properties of ELC-600 and ELC-700 After Stability Test**

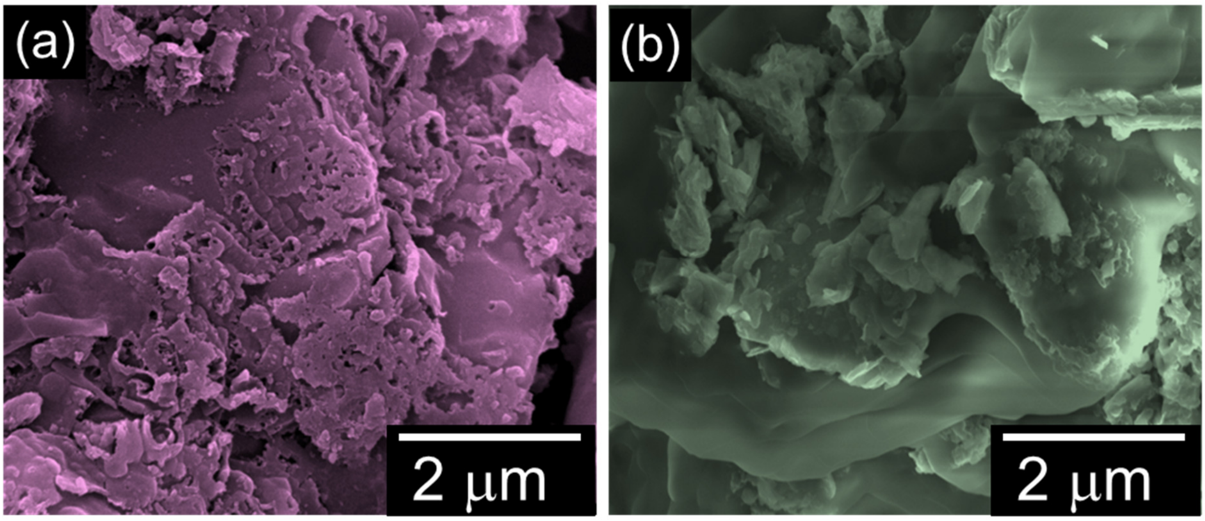

Figure S6: FE-SEM images of the (a) ELC-600 and the (b) ELC-700 catalysts after the stability test.

■ **Structural Properties of ELC-600 and ELC-700 After Stability Test**

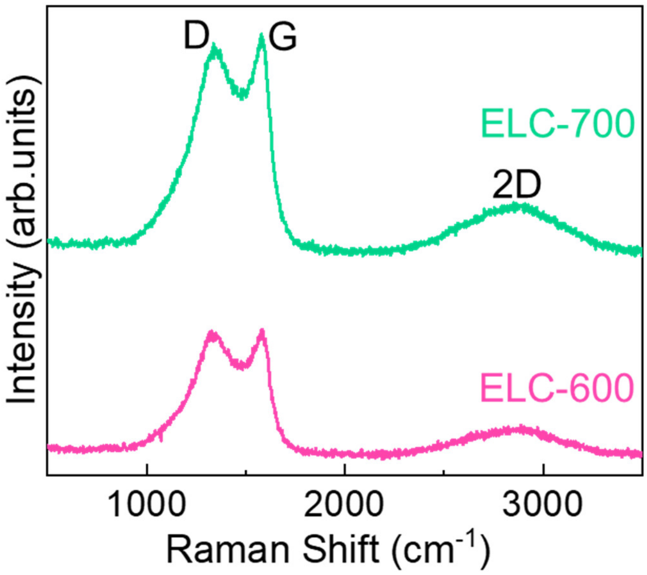

Figure S7: Raman spectra of the (a) ELC-600 and the (b) ELC-700 catalysts after the stability test.

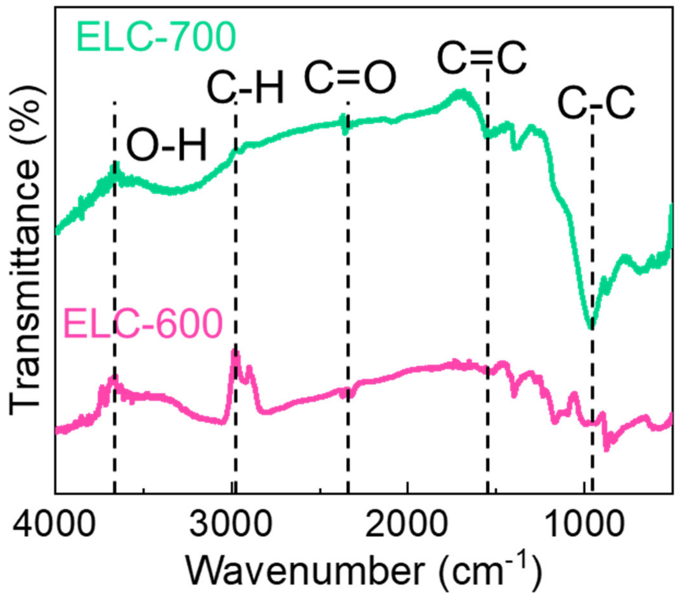

Figure S8: FTIR spectra of the (a) ELC-600 and the (b) ELC-700 catalysts after the stability test.

Table S1. Comparison of the HER performances between biomass ELC nanosheets and other carbonaceous electrocatalysts reported in previous works.

Catalyst	Activation Source	Morphology	Overpotential η (mV)	Tafel Slope S_T (mV/dec)	Electrolytes	References
**ELC-700**	**KOH**	**Agglomerated and interconnected sheets**	**39**	**36**	**0.5 M H_2SO_4**	**This Work**
**ELC-600**	**KOH**	**Aggregate stacked sheets**	**55**	**67**	**0.5 M H_2SO_4**	**This Work**
Nanoporous AC	KOH	2D sheets	380	85	0.5 M H_2SO_4	[1]
HH-AC-700	KOH	Aggregated Nanobundles	16	51	0.5 M H_2SO_4	[2]
Defective AC	NH_3 atmosphere	Graphitic layer	334	66	0.5 M H_2SO_4	[3]
Hierarchical porous carbon	KOH	Thin sheets	330	63	0.5 M H_2SO_4	[4]
N-doped AC	KOH	Stacked sheets	80	75	0.5 M H_2SO_4	[5]
N-doped Porous Carbon	Urea	Spongy	179	98	1 M KOH	[6]
Activated CNT	HNO_3	Nanotube	225	71	0.5 M H_2SO_4	[7]
Co, N co-doped carbon	Urea	Layered structure	223	91	0.5 M H_2SO_4	[8]

N- and S-codoped AC	Polystyrene sulfonic acid co-maleic acid Sodium Salt	Sponge	450	163	1 M KOH	[9]
N-, P- and Ca co-doped biochar	PVC + DMF	Hierarchical macro/mesoporous	309	87	0.5 M H_2SO_4	[10]
N- and S-codoped CNT	PDA + thiol	Nanotube	450	133	1 M KOH	[11]
N-doped Carbon Fiber	$KHCO_3$	3D network sheets	150	89	0.5 M H_2SO_4	[12]
N-doped carbon	HF etching	Particles	413	98	0.5 M H_2SO_4	[13]

References

1. Prabu, N., et al., *Bio-derived nanoporous activated carbon sheets as electrocatalyst for enhanced electrochemical water splitting.* Int. J. Hydrog. Energy, 2019. **44**(36): p. 19995-20006.
2. Sekar, S., D.H. Sim, and S. Lee, *Excellent Electrocatalytic Hydrogen Evolution Reaction Performances of Partially Graphitized Activated-Carbon Nanobundles Derived from Biomass Human Hair Wastes.* Nanomaterials, 2022. **12**(3): p. 531.
3. Yan, X., et al., *Activated carbon becomes active for oxygen reduction and hydrogen evolution reactions.* Chemical Communications, 2016. **52**(52): p. 8156-8159.
4. Prabu, N., et al., *An efficient palm waste derived hierarchical porous carbon for electrocatalytic hydrogen evolution reaction.* Carbon, 2019. **152**: p. 188-197.
5. Saravanan, K.R.A., et al., *Nitrogen-self doped activated carbon nanosheets derived from peanut shells for enhanced hydrogen evolution reaction.* Appl. Surf. Sci., 2019. **489**: p. 725-733.
6. Sathiskumar, C., et al., *Nitrogen-Doped Porous Carbon Derived from Biomass Used as Trifunctional Electrocatalyst toward Oxygen Reduction, Oxygen Evolution and Hydrogen Evolution Reactions.* Nanomaterials, 2020. **10**(1): p. 76.
7. Cui, W., et al., *Activated carbon nanotubes: a highly-active metal-free electrocatalyst for hydrogen evolution reaction.* Chemical Communications, 2014. **50**(66): p. 9340-9342.
8. Sun, H., et al., *Waste paper derived Co, N co-doped carbon as an efficient electrocatalyst for hydrogen evolution.* React. Kinet. Mech. Catal., 2021. **132**(2): p. 1137-1150.
9. Zhao, Y.-M., et al., *Activated carbon with heteroatoms from organic salt for hydrogen evolution reaction.* Microporous and Mesoporous Materials, 2020. **297**: p. 110033.
10. Wang, H., et al., *Solid Base Assisted Dual-Promoted Heterogeneous Conversion of PVC to Metal-Free Porous Carbon Catalyst.* Chem. Eur. J., 2022. **28**(23): p. e202200124.
11. Qu, K., et al., *Polydopamine-Inspired, Dual Heteroatom-Doped Carbon Nanotubes for Highly Efficient Overall Water Splitting.* Advanced Energy Materials, 2017. **7**(9): p. 1602068.
12. Liu, Y., et al., *Catalytically Active Carbon From Cattail Fibers for Electrochemical Reduction Reaction.* Front. Chem., 2019. **7**(786).
13. Cao, X., et al., *Synthesis of biomass porous carbon materials from bean sprouts for hydrogen evolution reaction electrocatalysis and supercapacitor electrode.* Int. J. Hydrog. Energy, 2021. **46**(36): p. 18887-18897.
